# Immune landscape of hepatocellular carcinoma: The central role of TP53-inducible glycolysis and apoptosis regulator

**DOI:** 10.1515/med-2024-0999

**Published:** 2024-07-30

**Authors:** Lingbing Qiu, Tianyi Ma, Yunmiao Guo, Jugao Chen

**Affiliations:** Department of Oncology, Shenzhen People’s Hospital, Second Clinical Medical College of Jinan University, First Affiliated Hospital of Southern University of Science and Technology, 518020, Shenzhen, Guangdong Province, P. R. China; Clinical Research Institute of Zhanjiang, Central People’s Hospital of Zhanjiang, Guangdong Medical University Zhanjiang Central Hospital, 236 Yuanzhu Road, 524045, Zhanjiang, Guangdong Province, P. R. China; Department of Oncology, Shenzhen People’s Hospital, Second Clinical Medical College of Jinan University, First Affiliated Hospital of Southern University of Science and Technology, No. 1017, Dongmen North Road, Luohu District, 518020, Shenzhen, Guangdong Province, P. R. China

**Keywords:** TP53-inducible glycolysis and apoptosis regulator, hepatocellular carcinoma, the cancer genome atlas, prognostic biomarker, gene set enrichment analysis, single-sample gene set enrichment analysis

## Abstract

**Objective:**

This study aims to address the substantive issue of lacking reliable prognostic biomarkers in hepatocellular carcinoma (HCC) by investigating the relationship between TP53-inducible glycolysis and apoptosis regulator (TIGAR) and HCC prognosis using The Cancer Genome Atlas database.

**Methods:**

(1) Integrated statistical analyses, including logistic regression, Wilcoxon signed-rank test, and Kruskal–Wallis test, were conducted to explore the association between TIGAR expression and clinical–pathological features of HCC. (2) The Kaplan–Meier method combined with univariate and multivariate Cox regression models underscored TIGAR as a prognostic factor in HCC. (3) Gene set enrichment analysis (GSEA) revealed key pathways associated with TIGAR, while single-sample gene set enrichment analysis (ssGSEA) determined its relevance to cancer immune infiltration.

**Results:**

(1) Elevated TIGAR expression was significantly correlated with decreased survival outcomes in HCC patients. (2) GSEA highlighted the significant link between TIGAR and humoral immunity. (3) ssGSEA revealed a positive correlation between TIGAR expression and infiltration of Th1 and Th2 cells and a negative correlation with Th17 cell infiltration.

**Conclusion:**

TIGAR, as a potential prognostic biomarker for HCC, holds significant value in immune infiltration. Understanding the role of TIGAR could contribute to improved prognostic predictions and personalized treatment strategies for HCC patients.

## 1 Introduction

Hepatocellular carcinoma (HCC) stands as the predominant histologic type of liver cancer, ranking as the third leading cause of cancer-related mortality globally [1]. Particularly prevalent in regions like Asia and Africa, chronic exposure to the hepatitis B virus remains its primary risk factor [2,3]. Despite notable therapeutic advancements in recent decades, our understanding of the molecular mechanisms driving its onset and progression remains substantially incomplete [4]. Furthermore, existing screening methodologies for HCC, including serum alpha-fetoprotein (AFP), ultrasonography, and CT scanning, often fail to detect the disease in its early stages. Consequently, most patients receive a diagnosis at an advanced stage, leading to dismal prognoses with a 5-year survival rate that does not exceed 20% [5]. This pressing scenario underscores an urgent need to identify viable biomarkers capable of facilitating early detection and targeted treatment for HCC patients.

Tumor suppressor TP53-inducible glycolysis and apoptosis regulator (TIGAR), a downstream target of P53, plays a pivotal role in apoptosis and autophagy regulation [6,7]. TIGAR inhibits glycolysis and the upstream genes of reactive oxygen species (ROS) responses, thereby modulating autophagy. This process can be disrupted by excessive nicotinamide adenine dinucleotide phosphate supplementation [7]. Moreover, TIGAR mediates glucose metabolism, regulating intracellular ROS levels to promote cell survival and DNA damage repair, highlighting its significance in these crucial cellular processes [8,9,10]. Studies have also hinted at TIGAR’s potential involvement in the regulation of cell cycle, proliferation, invasion, and metastasis-related proteins in tumor cells [11]. Noteworthy is the significant elevation of TIGAR expression observed in various human tumors such as colon cancer, breast cancer, and glioblastoma, suggesting its potential as a valuable tool in early tumor diagnosis and precise treatment [12,13,14]. However, the precise mechanisms governing HCC development and progression via TIGAR remain inadequately explored.

Therefore, our study aimed to bridge this knowledge gap, shedding light on the role of TIGAR in HCC and its prognostic implications. Our findings, particularly the association between elevated TIGAR expression and poorer survival rates in HCC patients, as well as the correlation between TIGAR and humoral immunity, have significantly expanded our understanding of the molecular landscape of HCC. Additionally, the discovery of distinct correlations between TIGAR expression and the levels of infiltrating Th1, Th2, and Th17 cells opens avenues for further investigation into the immunological mechanisms at play in HCC. From a clinical perspective, our results could revolutionize patient care. If TIGAR expression is confirmed as a reliable prognostic marker in HCC, it could be integrated into predictive models, enabling clinicians to forecast patient outcomes accurately and customize therapeutic strategies. Thus, our research signifies a significant stride toward more personalized and effective treatments for HCC patients.

## 2 Materials and methods

### 2.1 Data acquisition

We procured RNA-sequencing data (HTseq-counts) along with pertinent clinical data for 424 TCGA (The Cancer Genome Atlas)-LIHC samples from the TCGA database (https://portal.gdc.cancer.gov/). This dataset includes 50 samples from normal adjacent tissues and 374 from HCC tissues. Our analysis made use of the transcripts per million (TPM) read data from the same 424 subjects, with both normal and tumor tissues represented in this patient group.

The process encompassed the conversion of the TPM format from FPKM (fragments per kilobase per million), the application of log 2 transformation, and then using the resultant clinical and RNA-seq data for our research objectives. It should be noted that all the information deployed in this study was accessible to the public.

### 2.2 Differentially expressed gene (DEG) analysis

We employed the R package DEseq2 to distinguish DEGs among groups that exhibited distinct TIGAR expression levels, applying a cutoff of |log fold change (log FC)| ≥ 1.5 and a false discovery rate (FDR) of less than 0.05. To facilitate visualization of the differential expression analyses’ outcomes, we made use of R packages, namely EnhancedVolcano and pheatmap.

### 2.3 Enrichment analysis

We executed gene set enrichment analysis (GSEA) and gene ontology (GO) functional enrichment scrutiny using the R package known as Cluster Analyzer. To graphically represent the results, we employed the ggplot2 package in R. We set an FDR less than 0.25 and p.adjust <0.05 as the threshold for significant enrichment. For GO and Kyoto Encyclopedia of Genes and Genomes (KEGG) pathway enrichment, we used C5.mf.v7.2.symbols.gmt (GO) and C2.Cp.v7.2.symbols.gmt (Curated) as the reference gene sets, respectively. The categories considered in the GO analysis spanned cellular component (CC), molecular function (MF), and biological process (BP).

### 2.4 Protein–protein interaction (PPI) network

We constructed a PPI network of proteins co-expressed with TIGAR using the online database STRING (http://string-db.org). To do this, we fetched interacting proteins that showed an interaction score exceeding 0.4 and a PPI enrichment *P*-value <0.001. STRING is a digital platform dedicated to enabling the discovery of gene interactions. The procured TIGAR-associated proteins were subsequently imported into the Cluster Profiler 3.14.3 software, which facilitated the visualization of their mutual interaction patterns.

In addition, we carried out a differential examination of individual genes linked with TIGAR and scrutinized the co-expressed genes via enrichment analysis and KEGG pathway assessments. The output of these analyses was visually represented using the ggplot2 package.

### 2.5 Immune infiltration analysis

We harnessed the GSVA package [1.34.0] within the R environment [3.6.3] to execute single-sample gene set enrichment analysis (ssGSEA) on TIGAR, enabling a systematic review of immune infiltrates mentioned in extant literature. We explored the connection between TIGAR and signature genes associated with 24 distinct immune cell types.

To distinguish the differences in immune cell infiltration between TIGAR high-expression and low-expression groups, we employed Wilcoxon rank-sum tests. For the purpose of assessing the relationship between TIGAR gene expression and the density of tumor-infiltrating immune cells, we utilized mRNA sequencing data drawn from the TCGA Tumor Immune Estimation Resource (TIMER2.0) database (http://timer.cistrome.org/).

To establish the correlation between TIGAR and the 24 types of immune cells, we deployed the Spearman Correlation analysis. We used TIMER 2.0 to investigate the relationship between TIGAR expression and immune infiltration. This tool enables the estimation of immune infiltration based on the expression profiles provided by users using the CIBERSORT algorithm. The results were consistent with those obtained using the ssGSEA algorithm.

### 2.6 Statistical analysis

We processed statistical data obtained from TCGA utilizing the R package (version 3.6.3). The comparative analysis of TIGAR expression levels between HCC and normal groups was performed employing the Wilcoxon signed-rank test and Wilcoxon rank-sum test. The influence of Clinicopathological factors on TIGAR expression was evaluated through univariate logistic regression and Fisher’s exact tests.

We performed the Receiver Operating Characteristic (ROC) analysis of TIGAR using the Proc package (version 1.17.0.1). The area under the curve (AUC) was considered significant for our study if it ranged between 0.5 and 1. An AUC value closer to 1 symbolizes superior diagnostic efficacy, with a result above 0.9 signifying elevated accuracy. For all the statistical tests performed, a *P*-value <0.05 was regarded as significant.


**Ethics approval and consent to participate:** This article does not contain any studies with human participants or animals performed by any of the authors.

## 3 Results

### 3.1 Differential expression analysis of TIGAR in other cancers and HCC

**Figure 1:**
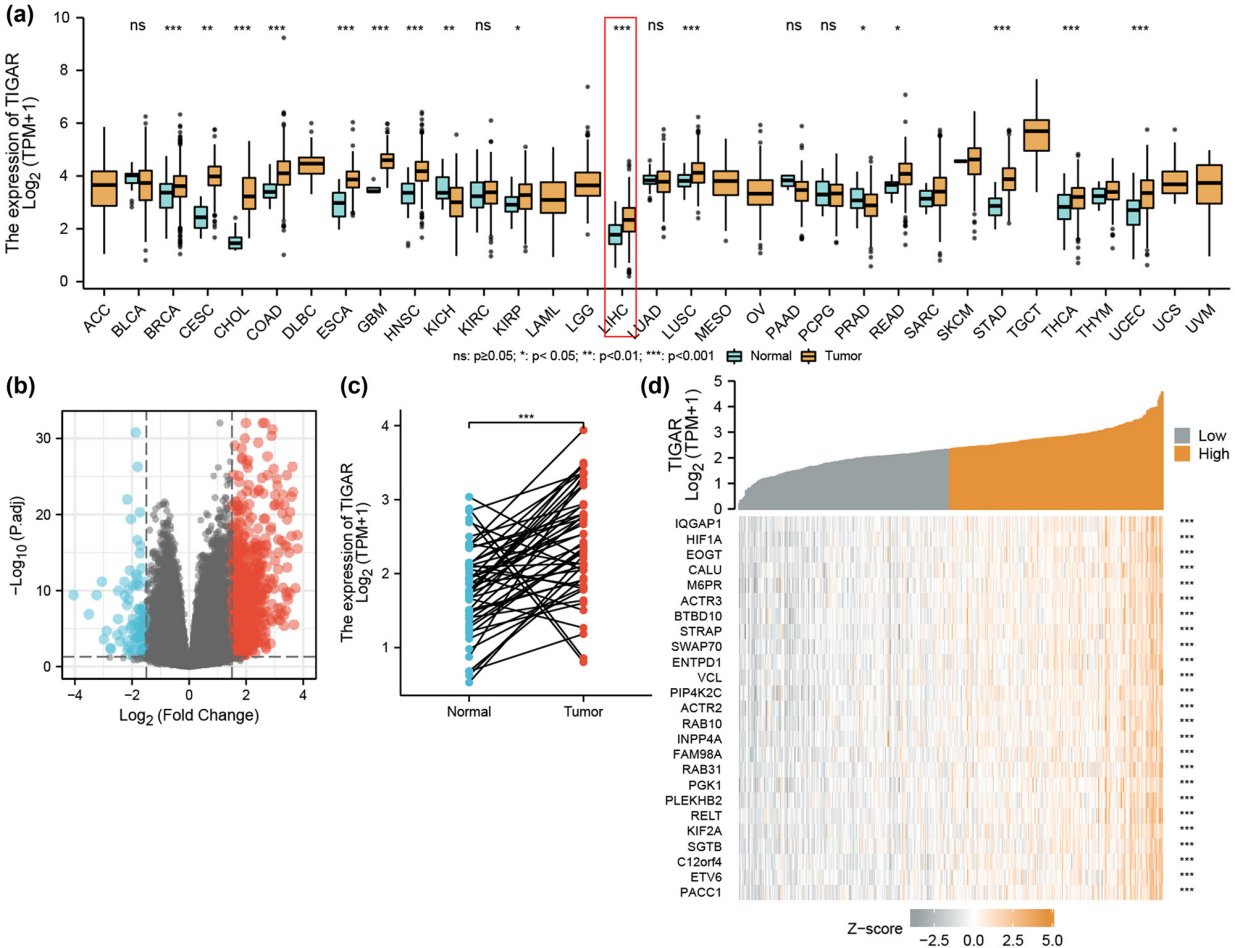
Differential expression levels of TIGAR in different malignancies and TIGAR-related DEGs. (a) Increased or decreased TIGAR of different cancers compared with normal tissues in the TCGA database. (b) The volcano plot of differentially expressed mRNAs. The red dots represent mRNAs with significantly high expression levels, blue dots represent mRNAs with significantly low expression levels, and gray dots indicate mRNAs with no significant differential expression. The selection criteria for significantly expressed mRNAs were set at |log fold change (logFC)| ≥ 1.5 and *p* < 0.05. (c) TIGAR expression level in case-matched HCC and normal tissues. (d) Volcano plots of the DEGs and heat map showing the 25 related genes (*** *p <* 0.01).

Gene expression analysis using the RNA-seq database revealed higher TIGAR mRNA expression in tumor samples compared to the corresponding normal tissues (*p* < 0.05) based on the Wilcoxon rank sum test (Figure 1a). These findings demonstrate that TIGAR expression is upregulated in cancer tissues. We investigated the expression of TIGAR in 50 normal samples and 374 HCC tumor samples derived from the TCGA dataset. Additionally, the volcano plots of TIGAR differentially expressed RNAs and the significantly high expression of TIGAR in HCC samples (*p* < 0.05) provide further support for our results (Figure 1b and c). Finally, the heat map illustrates 25 TIGAR-related genes (Figure 1d).

### 3.2 Functional enrichment analysis of DEGs with TIGAR

**Figure 2:**
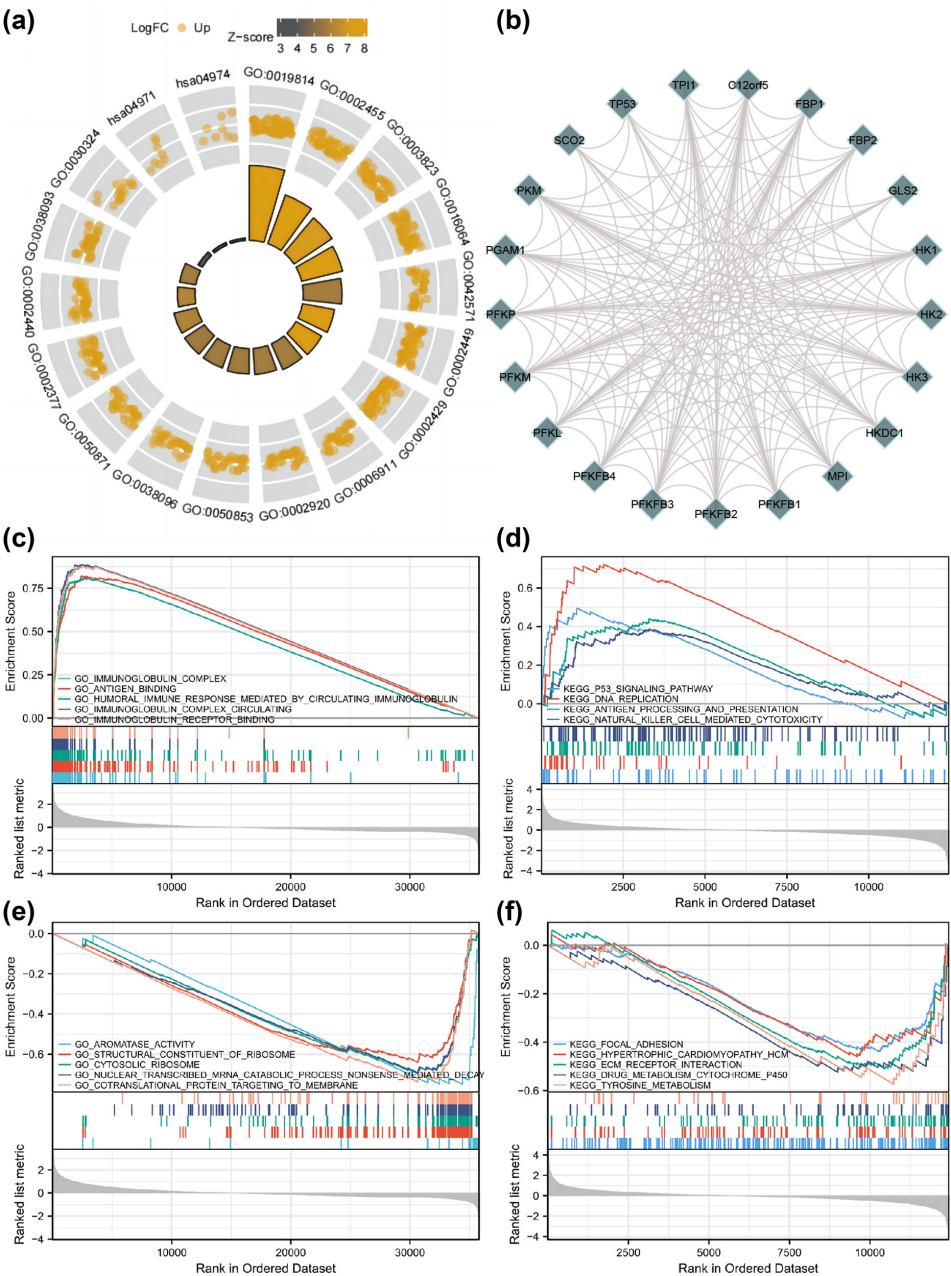
GO and KEGG enrichment analysis of TIGAR in HCC. (a) BP, CCs, MF enrichment, and KEGG related to TIGAR-related genes. (b) A network of TIGAR (TP53 protein) and 20 potential co-interaction proteins. (c)–(f) Results of enrichment analysis from GSEA.

The findings of the GO analysis revealed that TIGAR-related DEGs significantly regulate the CC, BP, and MF. Specifically, there were notable changes in the BP of TIGAR, which were associated with the humoral immune response mediated by circulating immunoglobulin, immunoglobulin-mediated immune response, lymphocyte-mediated immunity, and immune response-activating cell surface receptor signaling pathway, among others. The CC annotations involved immunoglobulin complex and circulating immunoglobulin complex, while the MF analysis indicated antigen binding (Figure 2a, Table 1). Furthermore, the PPI network of TIGAR demonstrated its potential co-expression proteins in TIGAR-related DEGs, as shown in Figure 2b. Specifically, TIGAR was found to be correlated with 21 genes such as TP53, TPI1, SCO2, and C12orf5. These genes consist of the Phosphofructokinase B Family (PFKFB1, PFKFB2, PFKFB3, and PFKFB4) and the Hexokinase Family (HK1, HK2, and HK3). Notably, TP53 and TPI1 have been linked to the inhibition of CD8 T cell infiltration and HCC metastasis [15,16]. Additionally, TCF19 and TP53 are involved in regulating TIGAR and SCO2 transcription in HCC, which is pivotal for mitochondrial energy metabolism and stress adaptation [17].

**Table 1:** TIGAR-related genes related to BP, CCs, MF enrichment, and KEGG

Ontology	ID	Description	*p*-value	*p*. adjust	*Z* score
BP	GO:0002455	Humoral immune response mediated by circulating immunoglobulin	5.51 × 10^−46^	9.32 × 10^−43^	7.211
BP	GO:0016064	Immunoglobulin-mediated immune response	7.71 × 10^−38^	5.86 × 10^−35^	7.280
BP	GO:0002449	Lymphocyte-mediated immunity	1.28 × 10^−29^	4.81 × 10^−27^	7.483
BP	GO:0002429	Immune response-activating cell surface receptor signaling pathway	4.57 × 10^−27^	1.40 × 10^−24^	7.810
BP	GO:0006911	Phagocytosis, engulfment	4.93 × 10^−26^	1.39 × 10^−23^	5.745
BP	GO:0002920	Regulation of humoral immune response	4.39 × 10^−24^	8.72 × 10^−22^	5.745
BP	GO:0050853	B-cell receptor signaling pathway	1.70 × 10^−23^	3.03 × 10^−21^	5.657
BP	GO:0038096	Fc-gamma receptor signaling pathway involved in phagocytosis	2.07 × 10^−22^	3.33 × 10^−20^	5.657
BP	GO:0050871	Positive regulation of B-cell activation	4.19 × 10^−22^	6.15 × 10^−20^	5.657
BP	GO:0002377	Immunoglobulin production	9.45 × 10^−22^	1.28 × 10^−19^	6.000
BP	GO:0002440	Production of molecular mediator of immune response	8.63 × 10^−17^	8.83 × 10^−15^	6.083
BP	GO:0038093	Fc receptor signaling pathway	7.78 × 10^−16^	7.74 × 10^−14^	5.745
BP	GO:0030324	Lung development	4.87 × 10^−06^	3.22 × 10^−04^	4.000
CC	GO:0019814	Immunoglobulin complex	1.41 × 10^−64^	4.91 × 10^−62^	8.124
CC	GO:0042571	Immunoglobulin complex, circulating	2.21 × 10^−34^	3.86 × 10^−32^	5.745
MF	GO:0003823	Antigen binding	9.90 × 10^−41^	5.32 × 10^−38^	7.000
KEGG	hsa04971	Gastric acid secretion	1.31 × 10^−04^	1.68 × 10^−02^	2.828
KEGG	hsa04974	Protein digestion and absorption	1.04 × 10^−03^	4.50 × 10^−2^	2.828

The GSEA analysis results for the GO term revealed a positive correlation between high TIGAR levels and signaling processes related to the immunoglobulin complex, antigen binding, and immunoglobulin receptor binding (Figure 2c). On the other hand, processes such as aromatase activity, structural constituent of ribosome, and cotranslational protein targeting to the membrane displayed a negative correlation with elevated TIGAR levels (Figure 2e).

Additionally, the curated gene set examination indicated a positive correlation between high TIGAR levels and the p53 signaling pathway, DNA replication, natural killer (NK) cell-mediated cytotoxicity, and antigen processing and presentation (Figure 2d). In contrast, focal adhesion, hypertrophic cardiomyopathy, hypertrophic cardiomyopathy extracellular matrix receptor interaction, drug metabolism cytochrome p450, and tyrosine metabolism were found to be negatively correlated with elevated TIGAR levels (Figure 2f). These observations propose a central enrichment of pathways regulating immune infiltrates and a robust association with TIGAR single-gene expression.

To deepen our understanding of TIGAR’s biological functions, we undertook GSEA on the variance between datasets reflecting low and high TIGAR expression. The aim was to identify GO and the GO term associated with TIGAR. The analysis of differentially expressed TIGAR genes unveiled a total of 678 KEGG pathways that showed noteworthy differential enrichment from the c5.all.v7.2.symbols.gmt (GO) dataset (FDR <0.05, adjusted *p* < 0.05). The KEGG pathways and GO term signatures most significantly enriched, based on their Normalized Enrichment Scores (NES), are presented in Table 2. The analysis revealed that TIGAR-related genes are primarily involved in BPs, such as the immune globulin complex, antigen binding, and humoral immune response mediated by circulating immunoglobulin. Additionally, these genes play a crucial role in pathways, including the p53 signaling pathway, DNA replication, drug metabolism cytochrome P450, and amino acid metabolism. Research has shown that diosgenin saponin exerts an effective role in HCC by modulating TIGAR-mediated cell apoptosis, autophagy, and DNA damage [18].

**Table 2:** Signaling pathways most significantly associated with TIGAR expression

Name	Description	NES	*p*-value	*p*. adjust
Positive GO term	GO_IMMUNOGLOBULIN_COMPLEX	3.394	0.001	0.009
	GO_ANTIGEN_BINDING	3.150	0.001	0.009
	GO_HUMORAL_IMMUNE_RESPONSE_MEDIATED_BY_CIRCULATING_IMMUNOGLOBULIN	3.093	0.001	0.009
	GO_IMMUNOGLOBULIN_COMPLEX_CIRCULATING	3.073	0.001	0.009
	GO_IMMUNOGLOBULIN_RECEPTOR_BINDING	3.042	0.001	0.009
Negative GO term	GO_AROMATASE_ACTIVITY	−2.636	0.003	0.013
	GO_STRUCTURAL_CONSTITUENT_OF_RIBOSOME	−3.369	0.017	0.044
	GO_CYTOSOLIC_RIBOSOME	−3.395	0.009	0.028
	GO_NUCLEAR_TRANSCRIBED_MRNA_CATABOLIC_PROCESS_NONSENSE_MEDIATED_DECAY	−3.397	0.011	0.031
	GO_COTRANSLATIONAL_PROTEIN_TARGETING_TO_MEMBRANE	−3.586	0.008	0.025
Positive KEGG term	KEGG_P53_SIGNALING_PATHWAY	1.982	0.003	0.029
	KEGG_DNA_REPLICATION	2.130	0.003	0.029
	KEGG_ANTIGEN_PROCESSING_AND_PRESENTATION	1.884	0.003	0.029
	KEGG_ANTIGEN_PROCESSING_AND_PRESENTATION	1.884	0.003	0.029
	KEGG_NATURAL_KILLER_CELL_MEDIATED_CYTOTOXICITY	1.803	0.003	0.029
Negative KEGG term	KEGG_FOCAL_ADHESION	−2.929	0.001	0.029
	KEGG_HYPERTROPHIC_CARDIOMYOPATHY_HCM	−1.663	0.005	0.038
	KEGG_ECM_RECEPTOR_INTERACTION	−1.731	0.003	0.029
	KEGG_DRUG_METABOLISM_CYTOCHROME_P450	−1.724	0.003	0.029
	KEGG_TYROSINE_METABOLISM	−2.858	0.002	0.029

### 3.3 Relationship between TIGAR expression and immune infiltration

**Figure 3:**
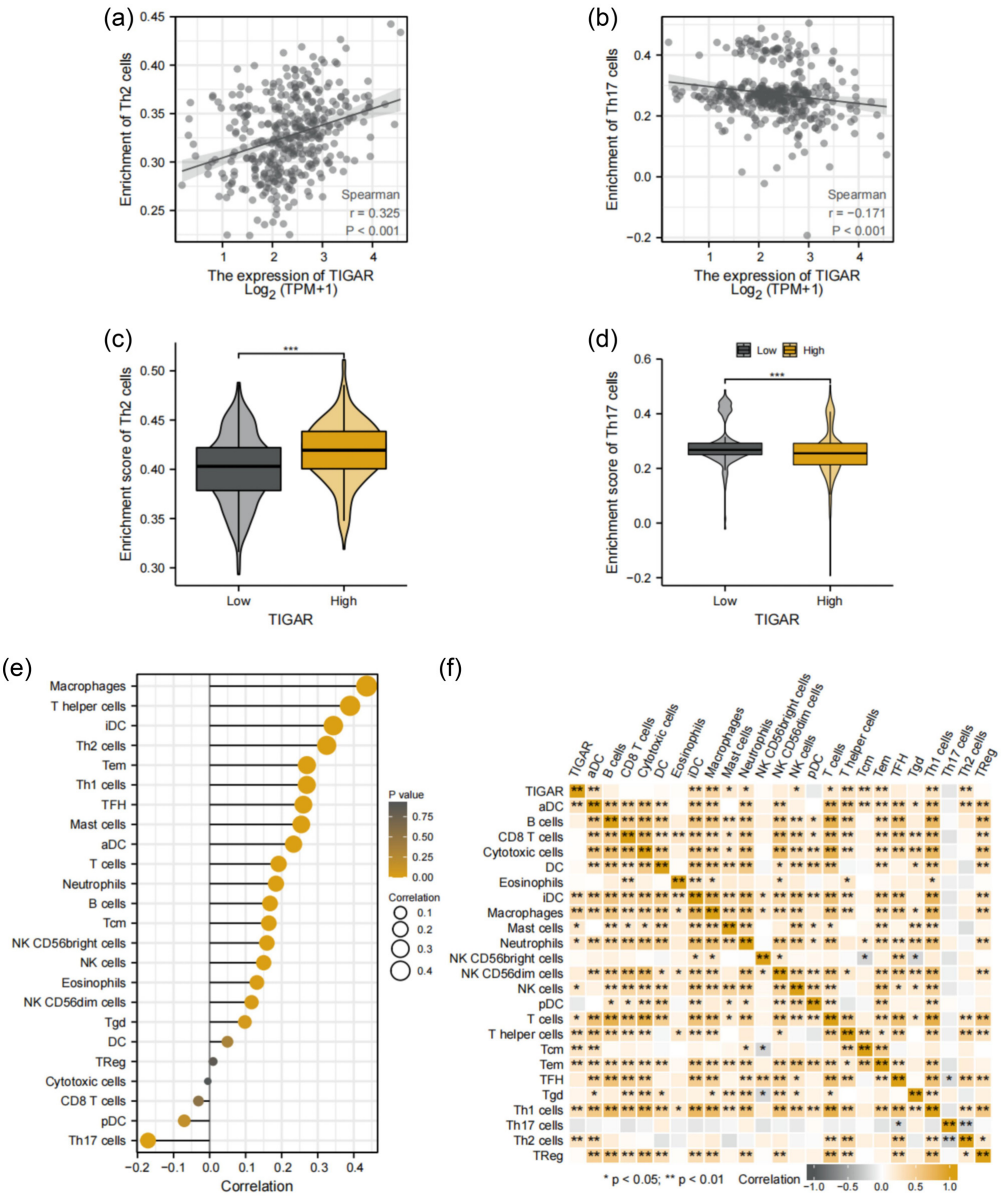
The results of analysis between TIGAR expression and immune infiltration. (a) The positive correlation between TIGAR expression and Th2 cells. (b) The negative correlation between TIGAR expression and Th17 cells. (c) Th2 cells’ infiltration level in different TIGAR expression groups. (d) Th17 cells’ infiltration level in different TIGAR expression groups. (e) and (f) Correlation between TIGAR expression level and the relative abundances of 24 immune cells.

Through the application of the Spearman correlation method, we examined the relationship between TIGAR expression levels and immune infiltration in the tumor microenvironment, using ssGSEA for our analysis. Interestingly, Th2 cells were observed to be significantly elevated in the high TIGAR expression cohort (*p* < 0.001), demonstrating a distinct positive correlation with TIGAR expression (Spearman *R* = 0.325, *p* < 0.001) (Figure 3a and c). In contrast, Th17 cells presented significantly lower levels in the high TIGAR expression cohort (*p* < 0.001) and exhibited a notable negative correlation with TIGAR expression (Spearman *R* = −0.171, *p* < 0.001) (Figure 3b and d).

To visually represent the correlation between TIGAR expression and the diversity of immune cells, we utilized lollipop plots. The derived results highlighted a positive association between TIGAR and various immune cells, including activated DC (aDC), B cells, immature DC (iDC), Macrophages, Mast cells, Neutrophils, NK CD56bright cells, NK cells, T cells, T helper cells, T central memory (Tcm), T effector memory (Tem), T follicular helper (Tfh), and Th1 cells. In contrast, Th17 cells exhibited a negative relationship with TIGAR (Figure 3e). Furthermore, a heat map provided visual evidence of the reciprocal relationship between TIGAR gene expression and the diverse types of tumor-infiltrating immune cells (Figure 3f). These findings emphasize the pivotal role that TIGAR undertakes in facilitating immune infiltration within HCC.

### 3.4 Associations between TIGAR expression and clinicopathological variables

**Figure 4:**
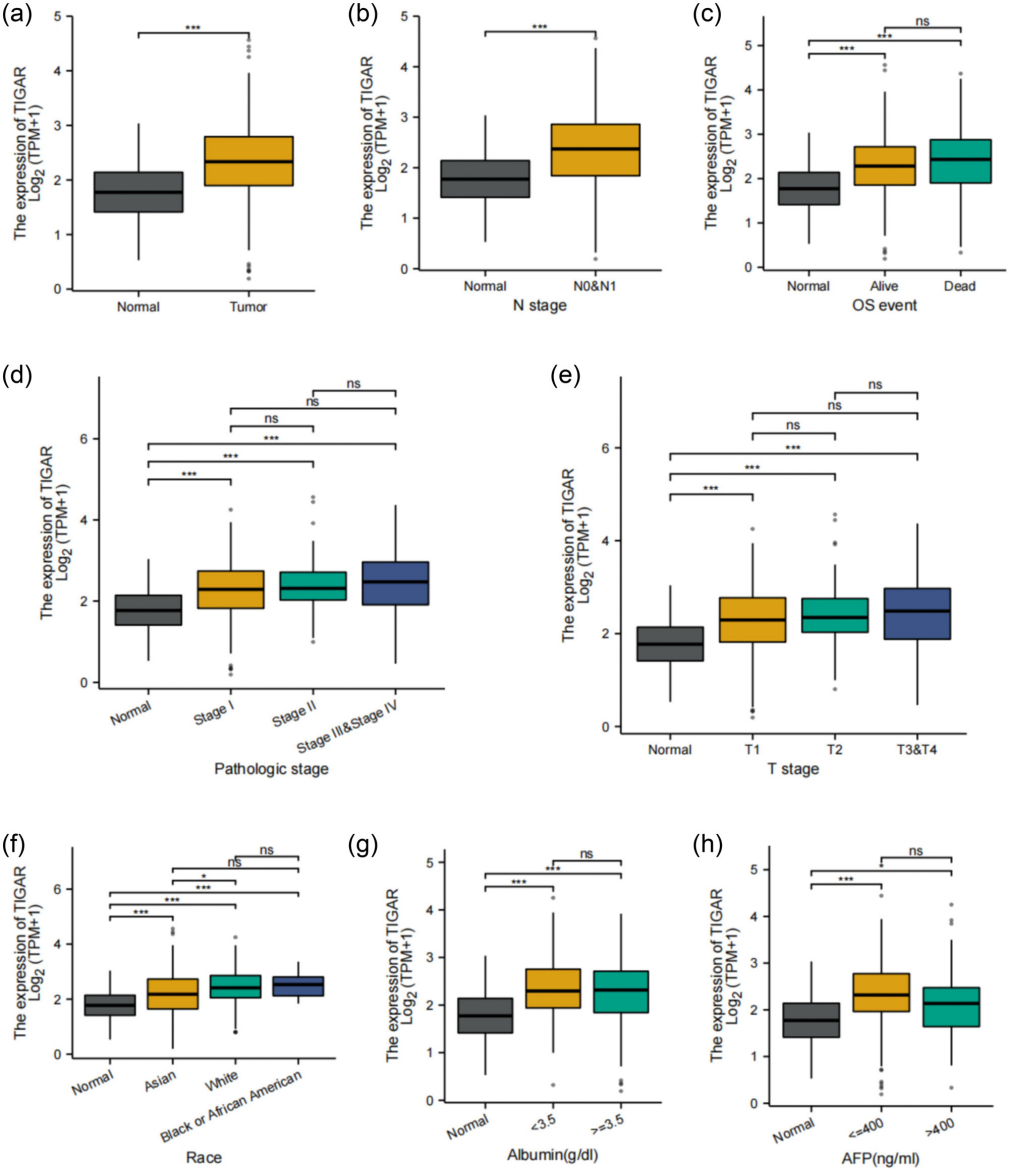
Association between TIGAR expression and different clinicopathological characteristics. (a) TIGAR expression between normal and tumor; (b) association between TIGAR expression and the N stage of HCC; (c) OS event; (d) the pathologic stage; (e) T stage; (f) race; (g) albumin; and (h) AFP.

Table 3 summarizes the correlation between TIGAR levels and clinicopathological characteristics, such as normal vs tumor, pathological N stage (N), pathological stage, pathologic stage, gender, age, neoplasm type, and tumor stage of LIHC patients (*n* = 374) from the TCGA-LIHC database. The expression of TIGAR shows significant differences among different patient characteristic groups, as illustrated in Figure 4a–h. Specifically, TIGAR exhibits higher expression in tumor samples and N0 and N1 samples compared to normal samples. Moreover, it is upregulated in Alive and Dead samples, as well as in Stage I, Stage II, pathologic stage, and Stage III and Stage IV samples. Additionally, TIGAR expression is elevated in T1, T2, and T3 and T4 samples. White patients show higher TIGAR expression compared to Asian patients. Furthermore, in samples with albumin levels <3.5 g/dl and albumin levels ≥ 3.5 g/dl, TIGAR expression is higher than in normal samples. Similarly, TIGAR expression is elevated in samples with AFP levels ≤400 ng/ml and AFP levels >400 ng/ml compared to normal samples.

**Table 3:** Clinicopathological characteristics of LIHC patients

Characteristics	Total (*N*)	Odds ratio (OR)	*p* value
T stage (T2 & T3 & T4 vs T1)	371	1.309 (0.871–1.972)	0.195
N stage (N1 vs N0)	258	0.939 (0.111–7.923)	0.950
M stage (M1 vs M0)	272	1.094 (0.130–9.224)	0.929
Pathologic stage (Stage III & Stage IV vs Stage I & Stage II)	350	1.414 (0.875–2.297)	0.159
Gender (male vs female)	374	1.130 (0.733–1.745)	0.581
Race (Black or African American & White vs Asian)	362	1.825 (1.202–2.784)	0.005
Age (>60 vs ≤60)	373	1.056 (0.703–1.586)	0.793
Weight (>70 vs ≤70)	346	1.517 (0.993–2.323)	0.055
Height (≥170 vs <170)	341	1.712 (1.110–2.654)	0.016
BMI (>25 vs ≤25)	337	1.221 (0.796–1.875)	0.361
AFP (ng/ml) (>400 vs ≤400)	280	0.744 (0.422–1.300)	0.302
Albumin (g/dl) (≥3.5 vs <3.5)	300	1.027 (0.599–1.764)	0.923
Prothrombin time (>4 vs ≤4)	297	1.772 (1.075–2.944)	0.026

Our findings highlight that TIGAR expression is elevated in LIHC tissues, with high TIGAR levels strongly correlating with LIHC progression and metastasis. Notably, logistic regression analysis underscored a significant association between TIGAR expression and the patient’s race (*p* = 0.005) (Table 4).

**Table 4:** TIGAR expression correlated with clinicopathological characteristics analyzed by logistic regression

Characteristics	Total (*N*)	Odds ratio (OR)	*p* value
T stage (T2 & T3 & T4 vs T1)	371	1.309 (0.871–1.972)	0.195
N stage (N1 vs N0)	258	0.939 (0.111–7.923)	0.950
M stage (M1 vs M0)	272	1.094 (0.130–9.224)	0.929
Pathologic stage (Stage III & Stage IV vs Stage I & Stage II)	350	1.414 (0.875–2.297)	0.159
Gender (male vs female)	374	1.130 (0.733–1.745)	0.581
Race (Black or African American & White vs Asian)	362	1.825 (1.202–2.784)	0.005
Age (>60 vs ≤60)	373	1.056 (0.703–1.586)	0.793
Weight (>70 vs ≤70)	346	1.517 (0.993–2.323)	0.055
Height (≥170 vs <170)	341	1.712 (1.110–2.654)	0.016
BMI (>25 vs ≤25)	337	1.221 (0.796–1.875)	0.361
AFP (ng/ml) (>400 vs ≤400)	280	0.744 (0.422–1.300)	0.302
Albumin (g/dl) (≥ 3.5 vs <3.5)	300	1.027 (0.599–1.764)	0.923
Prothrombin time (>4 vs ≤4)	297	1.772 (1.075–2.944)	0.026

**Figure 5:**
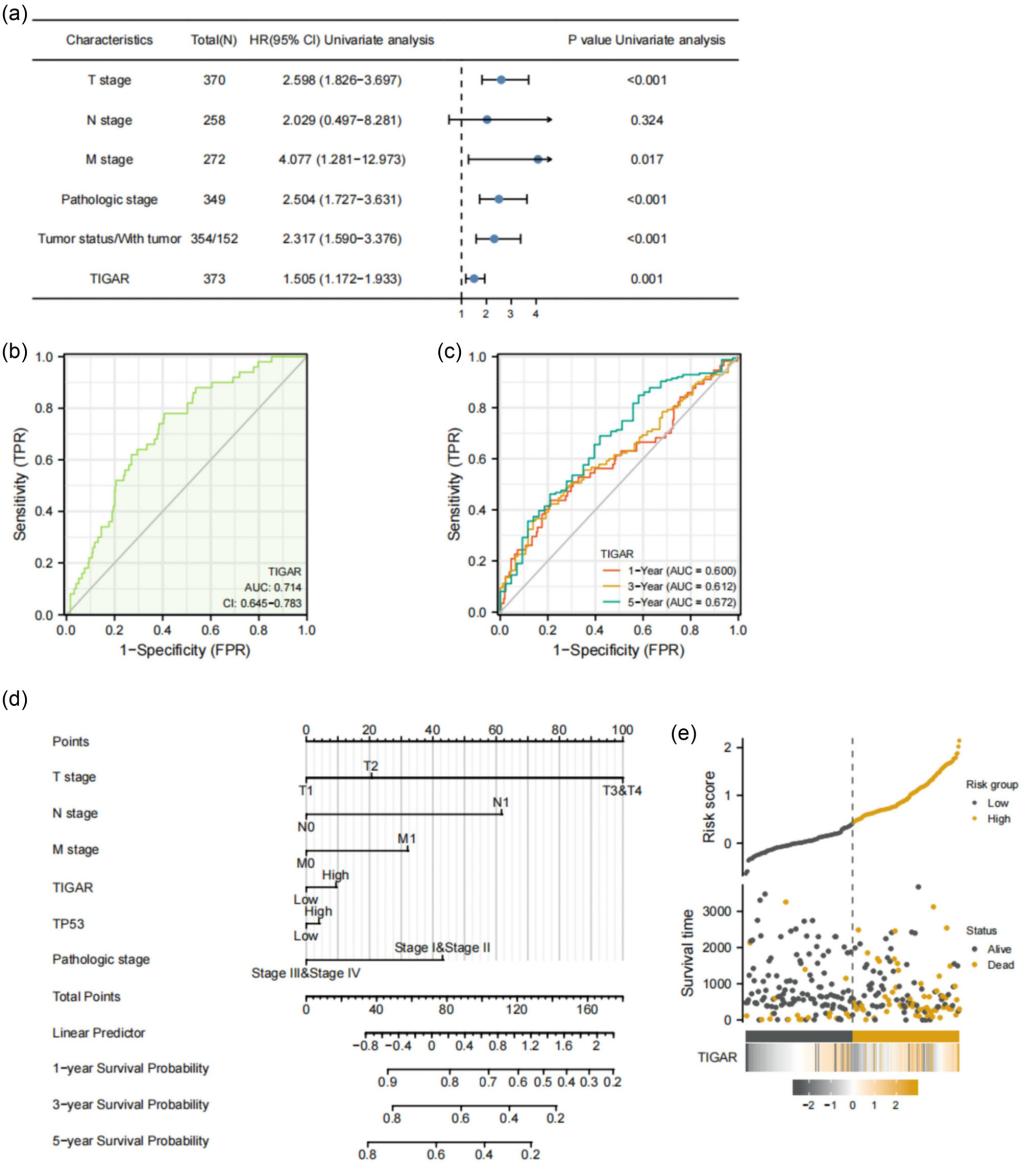
The prognostic value of TIGAR in LIHC. (a) Univariate Cox regression visualized in the forest plot. (b) Diagnostic ROC curve of TIGAR. (c) Time-dependent ROC curve of TIGAR. (d) A nomogram for predicting the probability of 1-, 3-, and 5-year OS for LIHC patients. (e) TIGAR expression distribution and survival status.

Univariate Cox regression analysis revealed that factors such as T stage (*p* < 0.001), M stage (*p* = 0.017), pathologic stage (*p* < 0.001), tumor presence (*p* < 0.001), and TIGAR expression (*p* < 0.001) are linked to poor prognosis for HCC (Figure 5a). Conversely, multivariate Cox regression identified tumor presence as a factor associated with worsened HCC prognosis (*p* = 0.004) (Table 5).

**Table 5:** Univariate and multivariate analyses of clinical pathological parameters in LIHC patients

Characteristics	Total (*N*)	Univariate analysis			Multivariate analysis	
		Hazard ratio (95% CI)	*p* value		Hazard ratio (95% CI)	*p* value
T stage	370					
T1 & T2	277	Reference				
T3 & T4	93	2.598 (1.826–3.697)	**<0.001**		1.586 (0.215–11.686)	0.651
N stage	258					
N0	254	Reference				
N1	4	2.029 (0.497–8.281)	0.324			
M stage	272					
M0	268	Reference				
M1	4	4.077 (1.281–12.973)	**0.017**		1.202 (0.286–5.052)	0.802
Gender	373					
Female	121	Reference				
Male	252	0.793 (0.557–1.130)	0.200			
Age	373					
≤ 60	177	Reference				
>60	196	1.205 (0.850–1.708)	0.295			
Race	361					
Asian	159	Reference				
White	185	1.323 (0.909–1.928)	0.144			
Black or African American	17	1.585 (0.675–3.725)	0.290			
Pathologic stage	349					
Stage I & Stage II	259	Reference				
Stage III & Stage IV	90	2.504 (1.727–3.631)	<0.001		1.452 (0.198–10.673)	0.714
Tumor status	354					
Tumor free	202	Reference				
With tumor	152	2.317 (1.590–3.376)	**<0.001**		1.975 (1.239–3.148)	**0.004**
TIGAR	373	1.505 (1.172–1.933)	**0.001**		1.355 (0.994–1.847)	0.054

Bold values represent the difference was statistically significant, less than 0.05.

In the univariate Cox analysis, the pathologic stage (*p* < 0.001), T stage (*p* < 0.001), and TIGAR expression were examined as prognostic indicators for overall survival (OS). After adjusting for covariates, the presence of a tumor (*p* = 0.004) emerged as a significant predictor of OS (Table 5). Given that the *p*-value for the multivariable analysis of TIGAR is 0.054, TIGAR demonstrates a marginal association with survival rates after adjusting for covariates. Intriguingly, the ROC analysis of TIGAR further validated the diagnostic precision of the score (AUC = 0.714, 90% CI: 0.645–0.783) (Figure 5b). Furthermore, we performed a reliable time-dependent ROC analysis to assess TIGAR’s time-dependent accuracy in predicting OS over 1, 2, and 3 years (Figure 5c). Finally, a nomogram was generated, facilitating quantitative prognosis prediction for LIHC patients by incorporating TIGAR and other independent clinical risk factors (Figure 5d).

Figure 5e demonstrates the distribution of TIGAR expression, the survival status of HCC patients, and the corresponding expression profiles of TIGAR. The findings indicate that increased TIGAR expression correlates with a worse prognosis. The gray and orange dots, respectively, denote surviving and deceased HCC patients. Importantly, the line on the upper left corresponds to the low-risk score group exhibiting low TIGAR expression, while the orange line on the right represents the high-risk score group showing high TIGAR expression. As the risk score in HCC patients escalates, the quantity of orange dots progressively increases, suggesting that high-risk group patients have lower survival rates and a higher likelihood of mortality.

**Figure 6:**
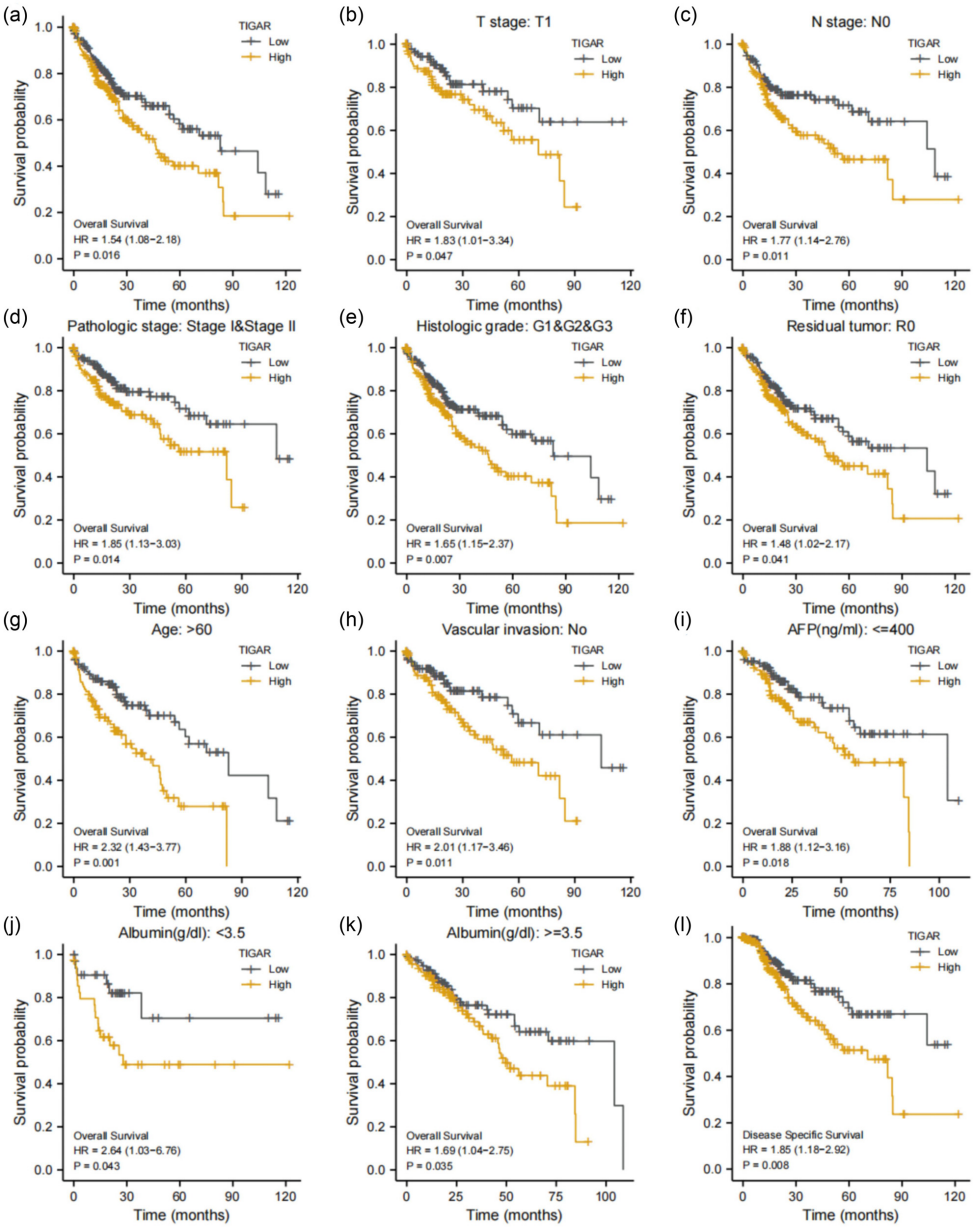
The prognostic value of TIGAR in the different subgroups. (a) The prognostic value of TIGAR in OS of HCC. (b)–(k) High expression of TIGAR associated with worse OS in different subgroups. (l) The prognostic value of TIGAR in DSS of HCC.

The prognostic value of TIGAR in HCC OS was evaluated using the Kaplan–Meier (K–M) survival curve, generated by the “survminer” R package. HCC patients were divided into high and low-expression groups based on the median value of TIGAR expression. The analysis revealed that the group with high TIGAR expression was significantly associated with poorer OS (HR = 1.54 (1.08–2.18), *p* = 0.016) (Figure 6a). Moreover, increased TIGAR expression was linked with poorer OS in various subgroups, including the T1 subgroup of T stage (HR = 1.83 (1.01–3.34), *p* = 0.047), stage N subgroup of pathologic stage (HR = 1.77 (1.14–2.76), *p* = 0.011), Stages I and II of pathologic stage (HR = 1.85 (1.13–3.03, *p* = 0.014)), G1 and G2 and G3 subgroup of histologic grade (HR = 1.65 (1.15–2.37), *p* = 0.007), and the R0 subgroup of tumor status (HR = 1.48 (1.02–2.17), *p* = 0.041) among others (Figure 6b–l).

## 4 Discussion

In the past decade, substantial progress has been achieved in the clinical diagnosis and treatment of HCC. This progress includes advancements in therapies such as surgical resection, chemotherapy, radiotherapy, molecule-targeted therapy, interventional therapy, immunotherapy, and traditional Chinese medicine [19,20]. Despite these multifaceted approaches, the prognosis for HCC patients remains dismal, with a 5-year survival rate of less than 20%, primarily due to a high rate of recurrence following treatment [21]. Previous studies have documented prognostic biomarkers for HCC, such as ALDOB, which is considered a prognostic biomarker for early diagnosis of HCC. Several inflammation-associated gene expression characteristics, including CD274 (PD-L1), CD8A, LAG3, and STAT1, are associated with improved objective response rates (*p* = 0.05) and OS (*p* = 0.01). These genes hold predictive value as biomarkers for HCC [22]. However, the predictive value of these biomarkers requires further confirmation in larger phase III trials. Consequently, there is an urgent need to identify potential biomarkers to improve the prognosis of HCC patients.

Abnormal tumor cell metabolism has been widely acknowledged in the field of oncology and has become a focal point of research. The concept traces back to 1924 when Warburg discovered that tumor cells could evade normal apoptosis processes through aberrant glucose metabolism. This abnormal metabolism, known as the Warburg effect, allows tumor cells to enhance proliferation and migration by generating ATP through aerobic glycolysis instead of the tricarboxylic acid cycle. The glucose metabolism status of tumor cells is closely linked to their survival and anti-apoptotic abilities. Notably, the TIGAR gene is a key player in tumor metabolism regulation. TIGAR, a downstream target of p53, regulates mitochondrial respiration and inhibits glycolysis, reducing ROS and increasing GSH levels, thus preventing tumor cell apoptosis [23]. TIGAR achieves this by modulating cellular redox levels, lowering F-2 and 6-P levels, or enhancing HK2 activity, allowing cells to evade apoptosis caused by ROS under stress. Moreover, TIGAR’s role in regulating autophagy in response to metabolic stress conditions is not fully understood. TIGAR expression is regulated through both p53-dependent and p53-independent mechanisms, altering the tumor metabolic microenvironment and promoting DNA repair and cell proliferation [24]. As an ROS restriction protein, TIGAR is highly expressed in tumor cells, exerting antioxidant effects by regulating NADPH production via the pentose phosphate pathway. This reduces apoptosis induced by oxidative damage and promotes tumor cell proliferation and metastasis [25]. The relationship between TIGAR and various solid tumors, including primary colon carcinoma, invasive breast carcinoma, lung cancer, nasopharyngeal carcinoma, gastric cancer, renal cell carcinoma, and prostate carcinoma, has been studied. High TIGAR expression is associated with aggressive tumor behavior and poor patient prognosis [26,27,28].

Tumor markers have become a key and effective method for early clinical diagnosis, and the combined detection of multiple markers can compensate for the limitations of single-marker testing, offering new strategies for the prognosis and auxiliary diagnosis of liver cancer [29]. In our research, we observed a significant upregulation of TIGAR expression in HCC tissue samples. This elevated TIGAR expression was associated with unfavorable survival outcomes in HCC patients, consistent with earlier studies [26,27,28]. Notably, the high-risk group exhibited substantially higher levels of TIGAR expression compared to the low-risk group. Utilizing a Cox regression model, we demonstrated that TIGAR, along with factors such as T stage, M stage, pathologic stage, and tumor presence, correlated with a poor prognosis for HCC. These findings suggest a pivotal role for TIGAR in HCC progression, urging further exploration of its functional dynamics within the disease. The findings presented above offer a theoretical basis for early detection of HCC. Monitoring the expression levels of TIGAR at different stages of pathological features can facilitate the development of more precise treatment strategies.

To delve deeper into TIGAR’s molecular mechanisms, we constructed co-expression networks associated with TIGAR in the context of HCC. Our analysis revealed that TIGAR co-expressed genes were predominantly involved in diverse processes, including the immunoglobulin complex, antigen binding, immunoglobulin receptor binding signaling, the p53 signaling pathway, DNA replication, NK cell-mediated cytotoxicity, and antigen processing and presentation. This intricate network underscores the multifaceted role of TIGAR in HCC, warranting comprehensive investigations to decode its functional dynamics within the disease.

Moreover, the significance of the tumor microenvironment in HCC progression has been increasingly acknowledged. The liver’s tumor microenvironment comprises various components, including tumor cells, tumor-infiltrating lymphocytes, tumor-associated macrophages, neutrophils, cancer-associated fibroblasts, myeloid-derived suppressor cells, dendritic cells, extracellular matrix, and other matrix-associated molecules [30]. The diverse distribution of tumor-infiltrating immune cells and the expression of immune checkpoint-related genes in HCC underscore the pivotal role of the tumor microenvironment in HCC progression.

CD8+ cytotoxic T lymphocytes play a crucial role in efficiently eliminating tumor cells through the secretion of cytokines associated with prognosis [31]. Conversely, regulatory T cells, a suppressive subset of CD4+ T lymphocytes, can inhibit immune responses initiated by CD8+ cytotoxic T lymphocytes, thereby promoting tumor evasion [32]. B cells exert anti-tumor effects by producing antibodies and acting as antigen-presenting cells to stimulate T-cell responses [33].

Upon activation by pharmaceuticals or other treatments, these lymphocytes have the potential to suppress HCC growth. Tumor-associated neutrophils can attract macrophages and regulatory T cells to tumor sites by releasing cytokines, thereby facilitating tumor progression and metastasis [34]. Certain molecular and CCs in the tumor microenvironment can induce functional abnormalities in dendritic cells, enabling tumor cells to evade immune detection [35]. In HCC, cancer-associated fibroblasts significantly contribute to tumor cell communication by activating various signaling pathways and expressing cytokines, establishing a microenvironment conducive to tumor cell growth [36].

Tumor-associated macrophages are classified into two phenotypes: the tumor-inhibiting M1 and tumor-promoting M2 [37]. M1-like macrophages can counteract HCC progression by modulating the tumor microenvironment, whereas M2-like macrophages can promote HCC cell proliferation and invasion by activating the TLR4/STAT3 signaling pathway [38,39]. Previous studies have reported that TIGAR increases during the formation of macrophage foam cells and atherosclerosis. Knocking down TIGAR significantly promotes lipid accumulation in macrophages [40]. Research has shown that desitabin downregulates TIGAR, inducing apoptosis and autophagy in myeloid leukemia cells, affecting cell metabolism, apoptosis, and immunity [41]. However, the relationship between TIGAR and macrophage polarization in HCC tumors has not been studied. In future research, we will continue to investigate this aspect further, aiming to explore its impact on the progression of HCC.

Our findings shed light on the complex interplay between TIGAR expression and immune cell infiltration within the tumor microenvironment, providing valuable insights into the mechanisms underlying HCC development and progression.

Our study revealed a compelling association between high TIGAR expression and diminished OS rates in HCC patients. Through GSEA, we have established a close relationship between TIGAR and humoral immunity, indicating the pivotal involvement of TIGAR in modulating the body’s antibody-mediated immune responses.

Furthermore, our analysis uncovered intriguing correlations between TIGAR expression and various immune cell populations. Specifically, Th17 cells, a subset of CD4+ T helper cells known for secreting inflammatory cytokines that promote tumorigenesis, exhibited significantly lower infiltration levels in the TIGAR high-expression group. This decrease in Th17 cell infiltration was notably correlated with the elevated expression of TIGAR, suggesting a negative regulatory role of TIGAR in Th17 cell-mediated inflammation within the HCC microenvironment. However, the effect size of Th17 is not too large. The biology that links TIGAR and Th17 needs further investigation.

Additionally, our study highlighted the intricate relationships between TIGAR and other immune cells, including aDCs, B cells, immature DCs, macrophages, mast cells, neutrophils, NK CD56 bright cells, NK cells, T cells, Tcm, Tem cells, and Tfh cells. These correlations underscore the multifaceted immunomodulatory roles played by TIGAR in shaping the immune landscape of HCC.

Macrophages, essential components of the immune system, play a crucial role in phagocytosing cellular fragments and pathogens. However, the polarization of macrophages may suppress the host’s immune response, potentially contributing to tumor development. In our analysis, we observed a complex interplay between TIGAR expression and macrophage polarization (Figure 3e and f), highlighting a possible mechanism through which TIGAR could influence immune responses within the HCC microenvironment.

Moreover, our study emphasized the pivotal role of NK cells, which are essential for initiating nonspecific immune responses and eliminating target cells, including various tumor cells. Elevated TIGAR expression levels were associated with altered NK cell activity, further implicating TIGAR in the dysregulation of immune surveillance against HCC cells. During the GO and GSEA analysis, several reasons may account for the lack of enrichment of glucose metabolism-related genes in the GO and GSEA entries. One potential explanation could be the threshold utilized for screening DEGs. Employing highly stringent thresholds may result in the exclusion of glucose metabolism-related genes, leading to their absence in the GO and GSEA analyses. Another possible reason could be attributed to the selection of the dataset, which may also elucidate the absence of significant glucose metabolism features in the analysis.

While our bioinformatics analysis has provided valuable insights, it is essential to acknowledge the limitations inherent in theoretical studies. To validate our findings and unravel the underlying mechanisms, further *in vivo* and *in vitro* experimental investigations are imperative. These experiments will not only corroborate our bioinformatics results but also offer a deeper understanding of the intricate molecular pathways through which TIGAR influences immune infiltration and HCC progression.

## 5 Conclusions

In summary, our study illuminates the intricate roles of TIGAR in HCC progression, emphasizing its impact on immune cell infiltration and overall patient survival. The correlations established between TIGAR expression and various immune cell populations underscore the complexity of immune regulation within the HCC microenvironment. While our bioinformatics analysis forms a solid foundation, future experimental studies are warranted to decipher the precise mechanisms through which TIGAR modulates immune responses and influences HCC development.
